# Comparison of Efficacy and Safety of Rosuvastatin, Atorvastatin and Pravastatin among Dyslipidemic Diabetic Patients

**DOI:** 10.1155/2013/146579

**Published:** 2013-02-10

**Authors:** Lolwa Barakat, Amin Jayyousi, Abdulbari Bener, Bilal Zuby, Mahmoud Zirie

**Affiliations:** ^1^Departments of Pharmacy and Clinical Pharmacy, Hamad General Hospital, Hamad Medical Corporation, P.O. Box 3050, Doha, Qatar; ^2^Departments of Medicine and Endocrinology, Hamad Medical Corporation, P.O. Box 3050, Doha, Qatar; ^3^Department of Medical Statistics and Epidemiology, Hamad Medical Corporation, P.O. Box 3050, Doha, Qatar; ^4^Departments of Public Health and Medical Education, Weill Cornell Medical College in Qatar, P.O. Box 3050, Doha, Qatar; ^5^Department of Evidence for Population Health Unit, School of Epidemiology and Health Sciences, The University of Manchester, Manchester, UK; ^6^Pediatric Intensive Care Unit, Department of Pediatrics, Hamad Medical Corporation, P.O. Box 3050, Doha, Qatar

## Abstract

*Objectives*. To investigate the efficacy and the safety of the three most commonly prescribed statins (rosuvastatin, atorvastatin, and pravastatin) for managing dyslipidemia among diabetic patients in Qatar. *Subjects and Methods*. This retrospective observational population-based study included 350 consecutive diabetes patients who were diagnosed with dyslipidemia and prescribed any of the indicated statins between September 2005 and September 2009. Data was collected by review of the Pharmacy Database, the Electronic Medical Records Database (EMR viewer), and the Patient's Medical Records. Comparisons of lipid profile measurements at baseline and at first- and second-year intervals were taken. *Results*. Rosuvastatin (10 mg) was the most effective at reducing LDL-C (29.03%). Atorvastatin reduced LDL-C the most at a dose of 40 mg (22.8%), and pravastatin reduced LDL-C the most at a dose of 20 mg (20.3%). All three statins were safe in relation to muscular and hepatic functions. In relation to renal function, atorvastatin was the safest statin as it resulted in the least number of patients at the end of 2 years of treatment with the new onset of microalbuminuria (10.9%) followed by rosuvastatin (14.3%) and then pravastatin (26.6%). *Conclusion*. In the Qatari context, the most effective statin at reducing LDL-C was rosuvastatin 10 mg. Atorvastatin was the safest statin in relation to renal function. Future large-scale prospective studies are needed to confirm these results.

## 1. Introduction

Diabetes is now commonly recognized as a “coronary heart disease risk equivalent” [1–4]. This is mainly attributed to the high rates of dyslipidemia among diabetic patients which is believed to be one of the major factors accounting for the high percentage of deaths among diabetics due to cardiovascular disease (CVD) [5].

The differences in the lipid profile between diabetics (especially type 2 diabetics) and nondiabetics account for the increased CVD risk [6]. Essentially, T2DM lipid profiles consist of elevations in triglyceride (TG) levels (>2 mmol/L) and reductions in high-density lipoprotein cholesterol (HDL-C). While low-density lipoproteins cholesterol (LDL-C) concentration levels are normal, the particles are denser and smaller in size, which is believed to enhance their atherogenic potential [7].

Numerous epidemiological studies and randomized controlled trials have documented the association between elevated LDL-C levels with increased CVD risk in both diabetic and nondiabetic populations [8, 9]. Thus reducing LDL-C levels is the primary goal of therapy for diabetic dyslipidemia [5, 10]. On the other hand, raising HDL-C and reducing triglycerides have been associated with moderate reductions in CVD risk and are thus regarded as of secondary importance [11].

Statins are considered the first pharmacological line of treatment of dyslipidemia in diabetic patients [12]. Lowering of LDL-C levels is thought to be the main beneficial effect of statin treatment; although, effects on HDL-C and other lipoproteins also play a role [5]. There are currently seven approved statins which are commonly prescribed, and each has a different benefit-risk profile [13].

To date, numerous randomized trials among various ethnicities have documented that rosuvastatin is the most effective statin at reducing LDL-C and triglycerides (TG) and at raising HDL-C levels [14, 15]. Atorvastatin, on the other hand, was previously, before the approval of rosuvastatin, documented to be the most potent statin at reducing LDL-C levels [16]. Alternatively, pravastatin which is available at the higher doses of 20 mg and 40 mg is found to be slightly less effective; the main reason for its prescription in patients is put down to its hydrophilic properties which make it more tolerable to patients with greater risk factors in addition to CVD [17].

Statins, like all other pharmacological treatments, inevitably have adverse side effects. The muscular system, hepatic function, and renal function have been documented to be affected by statin treatment [18, 19]. In general, large-scale randomized clinical trials have consistently demonstrated that statin therapy causes only a slight increased risk of side effects compared with placebo [20, 21]. For instance, postmarketing data report an overall adverse event frequency of less than 0.5% and a myotoxicity event rate of less than 0.1% [22].

In Qatar, there are currently no guidelines available for treating diabetic patients with dyslipidemia, and no previous study has documented the efficacy and safety of the various statins prescribed to diabetic patients. Thus the current study aims to build on this growing awareness of atherosclerosis-specific care of diabetes patients, by examining efficacy and safety of the three most commonly prescribed statins in the State of Qatar.

## 2. Methods

This retrospective observational population-based study was conducted in Hamad General Hospital, Hamad Medical Corporation which is the main tertiary hospital in Qatar serving, over 90% of the population of Qatar [23].

### 2.1. Study Patients

The study population was drawn from consecutive diabetic patients who presented to Hamad General Hospital with dyslipidemia and were prescribed the indicated statins and underwent fasting blood test of lipid profile before statin treatment initiation between September 2005 and September 2009. The diagnosis and when to treat dyslipidemia in diabetics in Hamad General Hospital are established on the basis of WHO expert group [24] criteria; the WHO recommends screening for lipid disorders at least annually in diabetic patients and more often if needed to achieve goals. On the other hand, adults with low-risk lipid values (LDL-C < 100 mg/dL [2.6 mmol/L], HDL-C > 50 mg/dL [1.3 mmol/L], and triglycerides < 150 mg/dL [1.7 mmol/L]) may be screened every two years.

The inclusion criteria included diabetic patients (fasting blood glucose ≥ 126 mg/dL [7.0 mmol/L]) who were prescribed any of the indicated statins (rosuvastatin, atorvastatin, pravastatin) during 2005–2009 (2 years within this period) to ensure at least 2 years of using the statin, aged ≥ 18 years, and had a total cholesterol level of ≥4 mmol/L, LDL-C ≥2.5 mmol/L, HDL-C ≤ 1 mmol/L in men and ≤1.2 mmol/L in women, and fasting triglycerides ≥ 1.7 mmol/L, obtained within 1 week before the first use of statins which will be then compared at first- and second-year intervals.

This study excluded pregnant women; patients with genetic disorders; patients on other concurrent lipid lowering agents such as bile acid sequestrants (cholestyramine, colesevelam), niacin, ezetimibe, fenofibrate and/or omega 3; patients with previous history of angina, severe vascular disease, or other life threatening disease; patients with nephropathy and/or hypothyroidism, active liver disease, bile duct problems, or ALT > 3 × ULN; patients with creatine kinase levels > 10 × ULN; patients taking concurrent corticosteroids, ciclosporin, and/or hormone replacement therapy; patients who are physically inactive; patients with a history of drug or alcohol abuse. 

IRB ethical approval, for this study, was obtained from Medical Research Committee of Hamad Medical Corporation prior to commencement of data collection.

### 2.2. Data Collection

Patients were identified as statin users (rosuvastatin, atorvastatin, pravastatin) during the study period (September 2005–September 2009) by using the Pharmacy Database. Data collection sheets were designed by the researcher for each patient. Fifty patients were included in the study for each statin dose, and these had to have been on the same dose for at least 2 years between the periods indicated previously. The data for these patients were obtained from 2 sources which are the electronic medical database called (EMR viewer) and the patients' files from the Medical Records Department of Hamad Medical Corporation (HMC). The prescribed doses in HMC for diabetic patients with dyslipidemia are as follows:

- rosuvastatin 10 mg and 20 mg,
- pravastatin 20 mg and 40 mg,
- atorvastatin 10 mg, 20 mg, and 40 mg.

Patients had been checked 3 times to exclude duplications which finally resulted in a total sample of 350 patients.

### 2.3. Data Collection Sheet

The researcher developed a structured data collection sheet for this study. The measures consisted of questions relating to sociodemographic data, medical history, and comorbid factors, lifestyle habits, lab investigations, and patient's antihypertensive medications (ARBs and/or ACE inhibitors). The designed data collection sheet was tested among 25 patients as a pilot study. Thereafter, any discrepancies found between the data collection sheet and the data available in the Medical Record were resolved.

The first part included the type of statin prescribed for the patient for at least 2 years continuously and dose of statin. The second part included information about sociodemographic characteristics including age, sex, nationality, height, weight, and BMI. Lifestyle habits like smoking and alcohol intake were included in the second section. The third section collected information about type of DM, its duration, and presence of hypertension. The fourth section included items about laboratory investigations such as fasting blood glucose, glycated hemoglobin (HbA1C), total cholesterol, HDL and LDL cholesterol levels, triglycerides, creatine kinase level, serum creatinine, bilirubin, LFTs, GGT, and serum albumin and adverse events like microalbuminuria and macroalbuminuria. Finally the fifth section included the initiation of antihypertensive medications (ARBs and/or ACE inhibitors). 

### 2.4. Statistical Analysis

Data were analyzed using SPSS version 20. Student's *t*-test was used to ascertain the significance of differences between mean values of two continuous variables and confirmed by nonparametric Mann-Whitney test. In addition, paired *t*-test was used to determine the difference between baseline and 2 years after regarding biochemistry parameters, and this is confirmed by the Wilcoxon test which is a nonparametric test that compares two paired groups. Chi-square and Fisher exact tests were performed to test for differences in proportions of categorical variables between two or more groups. The level *P* < 0.05 was considered as the cutoff value or significance.

## 3. Results

Table 1 presents the sociodemographic characteristics of the study population. The majority of patients were Qatari, had a BMI > 30 kg/m^2^, and had comorbid hypertension.

**Figure 1:**
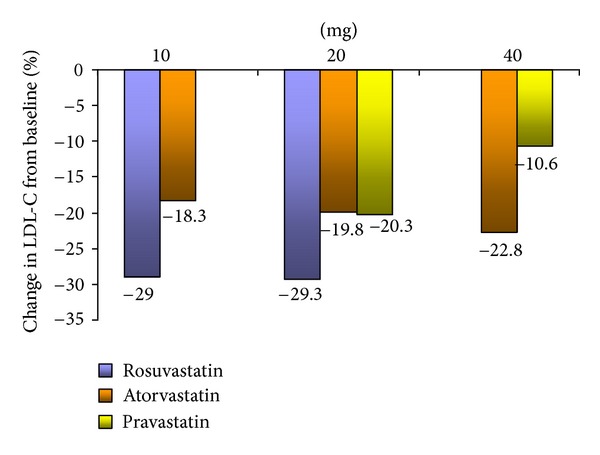
(%) Change in LDL-C from baseline.

**Figure 2:**
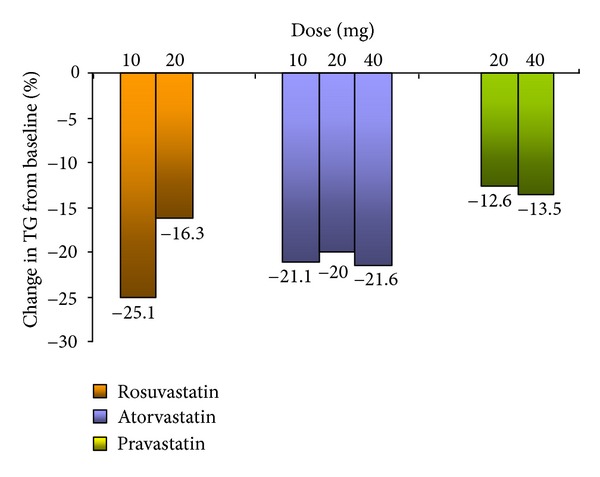
Rosuvastatin versus other statins, change in triglycerides.

Table 2 and Figures 1 and 2 show the comparison of the efficacy of each statin at each dose (10 mg, 20 mg, and 40 mg). Rosuvastatin (10 mg and 20 mg) was the most effective at reducing LDL-C (29.03% and 29.3%, resp.). Atorvastatin reduced LDL-C the most at a dose of 40 mg (22.8%), and pravastatin reduced LDL-C the most at a dose of 20 mg (20.3%). Moreover, rosuvastatin (10 mg) reduced triglycerides the most (−25.1%, *P* < 0.01). This was the case, even in comparison to higher doses of rosuvastatin (20 mg) and to higher doses of atorvastatin (20 mg and 40 mg) and pravastatin (20 mg and 40 mg). Finally, all of the statins appear to have reduced rather than raised HDL-C levels.

**Figure 3:**
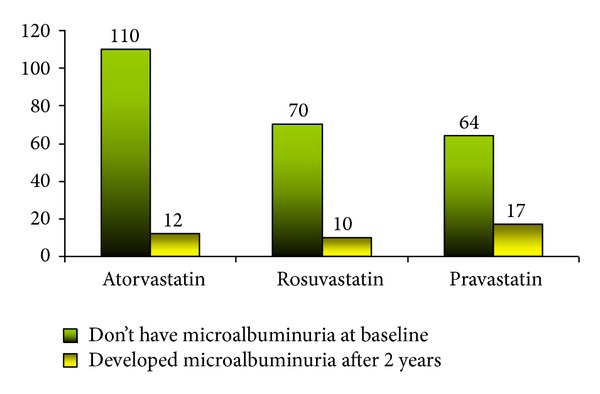
Number of patients with no microalbuminuria at baseline but developed it after 2 years.

Figure 3 presents the percentage of patients with the new onset of microalbuminuria after taking statins. Atorvastatin was the safest statin as it resulted in the least number of patients at the end of 2 years of treatment with the new onset of microalbuminuria (10.9%) followed by rosuvastatin (14.3%) and then pravastatin (26.6%).

Table 3 presents an overview of the safety profile of each of the statins at their various doses among patients. Statins appear to be safe in relation to hepatic and muscular functions as no patients presented with ALT > 3 × ULN or CK > 10 × ULN. A slightly adverse effect of statins on renal function was observed due to the new onset of microalbuminuria among some of the patients; nonetheless no case of microalbuminuria progressed to the more dangerous macroalbuminuria.

## 4. Discussion

In the present study, rosuvastatin (10 mg and 20 mg) was found to be the most effective statin at reducing LDL-C when compared with atorvastatin (10 mg, 20 mg, and 40 mg) and pravastatin (20 mg and 40 mg). In other words, rosuvastatin at its lowest dose in this study (10 mg) was more effective at reducing LDL-C levels than atorvastatin and pravastatin at their highest doses (40 mg). Indeed, it should be noted that rosuvastatin, which is the latest statin to receive approved labeling by the Food and Drug Administration, has been consistently found to be the most effective at reducing LDL-C levels in the most recent studies comparing its efficacy to other statins [25]. Our results are consistent with STELLAR trial which is one of the major open-label, randomized, and multicenter trials to compare rosuvastatin (10, 20, 40, or 80 mg) with atorvastatin (10, 20, 40, or 80 mg), pravastatin (10, 20, or 40 mg), and simvastatin (10, 20, 40, or 80 mg) across dose ranges for reduction of LDL-C [15]. The results of the STELLAR trial revealed that rosuvastatin was consistently, across all doses, the most effective at reducing LDL-C levels in comparison to all of the other statins.

The lowering of triglycerides is another important goal in reducing CVD risk among diabetic patients [5]. In the current study, the greatest reduction in triglycerides was (−25.1%, *P* < 0.01) and was achieved by patients taking rosuvastatin (10 mg). This was the case, even in comparison to higher doses of rosuvastatin (20 mg) and to higher doses of atorvastatin (20 mg and 40 mg) and pravastatin (20 mg and 40 mg). However, it is important to note that atorvastatin (10 mg and 40 mg) both achieved the second highest reduction in triglycerides (−21.05%, *P* < 0.05, and −21.56%,  *P* = NS), respectively. These findings are similar to the majority of studies in the literature, which have shown a slightly higher reduction in triglycerides in patients taking rosuvastatin in comparison to atorvastatin [26]. It thus appears that, in relation to this factor (triglycerides), that both rosuvastatin and atorvastatin are effective at reducing it.

Raising HDL-C levels is another major factor known to reduce CVD risk [11, 27]. In the current study, all of the statins appear to have reduced rather than raised HDL-C levels. Rosuvastatin (20 mg) had the least reduction of (−4.0%) and would thus be regarded as the most effective; however, none of the values for the statins were significant.

In this instance, the findings in the current study are contrary to the studies in the literature. For example, the STELLAR trial [15] found rosuvastatin (40 mg) to be the most effective on increasing HDL-C. In fact it is noted that across dose ranges, the HDL cholesterol increasing effect of rosuvastatin was consistent across the dose range and was significantly higher (*P* < 0.001) compared with those of simvastatin and pravastatin [15]. As for the PULSAR [26] study which investigated starting doses of rosuvastatin and atorvastatin, they found that the increase in HDL-C was significantly greater statistically with rosuvastatin (10 mg) than with atorvastatin (20 mg).

A number of factors could be at play which could explain the discrepancy between the current study's results and those in the international literature. It is evident from Table 1 which has the patient characteristics that many of the patients had more than two risk factors for CVD. For instance, the patients were diabetic, and most of them were over 55 years old and had hypertension. In addition, over two-thirds of the patients were obese and Qatari. In other diabetic research studies conducted in Qatar, it was found that having Qatari ethnicity was correlated with poor lifestyle habits such as sedentary lifestyle and poor eating choices [2, 3]. In other words, the subjects had so many other factors which caused their HDL-C levels to be so low that the statins were only able to have a minimal effect.

One of the most common complaints related to statin use is related to the effect of statins on muscular function. Muscle symptoms range from myalgia, which includes muscle pain without creatine kinase (CK) elevations, to myositis which is muscle symptoms with CK elevations [18]. In general, elevations of CK of more than ten times the upper limit of normal are regarded as significant elevations justifying the discontinuation of statin treatment [28]. In the current study, there were no cases of elevations of CK level > 10 × ULN. In other words, all statins, irrespective of dose, were regarded as safe in terms of myositis.

Hepatic function is also known to be affected by statin use [18]. This is mainly measured by asymptomatic elevations of the liver enzymes ALT and AST, otherwise known as transaminitis [29]. In the current study, no patients had elevated ALT > 3 × upper limit of normal. Thus there were no adverse events related to hepatic function reported with the use of any of the statins in the study. This is not surprising, as clinical trials have reported a 0.5–3.0% occurrence of elevations in aminotransferases among patients receiving statins and very rare episodes of severe liver injury. In general, the incidence of hepatic failure in patients taking statins appears to be in different from that in the general population [29].

In the current study, the three statins (atorvastatin 10, 20, and 40 mg; rosuvastatin 10 and 20 mg; and pravastatin 20 and 40 mg) with different doses did not significantly affect the serum creatinine and GFR after 2 years. In addition, the three statins appeared to be relatively safe for patients with microalbuminuria at baseline, as the number of those whose microalbuminuria increased was very minimal. In fact, pravastatin (40 mg) appeared to reduce the number of patients with baseline microalbuminuria. On the other hand, in patients without baseline microalbuminuria, there appeared to be relatively significant onset of microalbuminuria in patients taking pravastatin (26.6%) and to a lesser degree rosuvastatin (14.3%) and atorvastatin (10.9%). The literature relating to the effect of statins on microalbuminuria is somewhat controversial. While some statin trials have reported a reduction in proteinuria [30] or no effect [31], some of the literature supports the findings in the current study that statins do have negative effects relating to the onset of proteinuria [19]. The main reason stated for atorvastatins' safety in relation to renal function is its somewhat unique method of metabolism; in that it has the least degree of renal excretion (2%), followed by fluvastatin (5%), rosuvastatin (10%), lovastatin (10%), simvastatin (13%), and pravastatin (20%) [32].

## 5. Conclusion

The results obtained in the current study have shown that rosuvastatin is the most effective statin at reducing LDL-C, triglycerides, and total cholesterol, at the lowest dose (10 mg). Moreover, it reduced HDL-C the least in comparison to the other statins. Thus in the Qatari diabetic dyslipidemic population it appears to be the most effective statin. In addition, this study provides a good base for future large-scale prospective studies to be conducted on the topic.

## Figures and Tables

**Table 1:** Socio-demographic characteristics of study population (*n* = 350).

Variables	Type of Statin		
	Atorvastatin (*n* = 150) *n* (%)	Rosuvastatin (*n* = 100) *n* (%)	Pravastatin (*n* = 100) *n* (%)
Age (Mean ± SD)	57.4 ± 12.7	55.8 ± 9.8	57.5 ± 10.3
Age group			
<65 years	117 (78.0)	80 (80.0)	76 (76.0)
≥65 years	33 (22.0)	20 (20.0)	24 (24.0)

Nationality			
Qatari	110 (73.3)	69 (69.0)	79 (79.0)
Non-Qatari	40 (26.7)	31 (31.0)	21 (21.0)
Gender			
Male	54 (36.0)	55 (55.0)	34 (34.0)
Female	96 (64.0)	45 (45.0)	66 (66.0)

BMI (Mean ± SD)	33.6 ± 8.3	31.9 ± 6.4	33.3 ± 7.1

BMI group			
<25 kg/m^2^	10 (6.7)	11 (11.0)	8 (8.0)
25–30 kg/m^2^	40 (26.7)	36 (36.0)	23 (23.0)
≥30 kg/m^2^	100 (66.7)	53 (53.0)	69 (69.0)

Smokers			
Yes	19 (12.7)	17 (17.0)	12 (12.0)
No	131 (87.3)	83 (83.0)	88 (88.0)

Alcohol consumer			
Yes	2 (1.3)	1 (1.0)	5 (5.0)
No	148 (98.7)	99 (99.0)	95 (95.0)

HTN			
Yes	130 (86.7)	95 (95.0)	94 (94.0)
No	20 (13.3)	5 (5.0)	6 (6.0)

DM			
Type I	6 (4.0)	7 (7.0)	—
Type II	144 (96.0)	93 (93.0)	100 (100)

DM duration			
<5 years	17 (11.3)	18 (18.0)	9 (9.0)
5–10 years	47 (31.3)	35 (35.0)	35 (35.0)
>10 years	86 (57.3)	47 (47.0)	56 (56.0)

*Continuous variables are presented as mean ± SD and categorical variables as numbers (percentages).

**Table 2:** Comparison of the efficacy of statins for all doses (10 mg, 20 mg, and 40 mg).

	Ator 10 mg	Ator 20 mg	Ator 40 mg	Ros 10 mg	Ros 20 mg	Prav 20 mg	Prav 40 mg
% reduction LDL	−18.29^†^	−19.84^†^	−22.81^†^	−29.03^†^	−29.3^†^	−20.27^†^	−10.6^‡^
% reduction TG	−21.05^‡^	−19.95^*ξ*^	−21.56	−25.1^*ξ*^	−16.72	−12.63	−13.5
% reduction total cholesterol	−16.65^†^	−13.95^†^	−17.51^†^	−22.42^†^	−26.18^†^	−15.91^†^	−15.91^†^
% increase HDL	−7.21	−5.56	−6.49^*ξ*^	−6.64	−4.0	−5.88	−9.97
(%) patients meeting *European LDL-C goals after 2 years	60.0	44.0	50.0	56.0	58.0	48.0	32.0
(%) total cholesterol < 4 mmol/L after 2 years	40.0	32.0	19.6	38.0	44.0	30.0	24.5
(%) patients TG < 1.7 mmol/L after 2 years	64.0	56.0	39.2	58.0	54.0	50.0	55.1
(% ) patients HDL > 1 mmol/L after 2 years	70.0	74.0	68.6	66.0	65.3	68.0	74.0

*LDL-C < 2.5 mmol/L (100 mg/dL) for patients with CVD and/or type 2 diabetes.

^†^ *P* value ≤ 0.001, ^*ξ*^ *P* value ≤ 0.01, and ^‡^ *P* value ≤ 0.05.

**Table 3:** Safety comparison on hepatic, renal, and muscular functions: adverse events after 2 years of statin use.

Variables	Ator 10 mg *n* = 50 (%)	Ator 20 mg *n* = 50 (%)	Ator 40 mg *n* = 50 (%)	Ros 10 mg *n* = 50 (%)	Ros 20 mg *n* = 50 (%)	Prav 20 mg *n* = 50 (%)	Prav 40 mg *n* = 50 (%)
Hepatic function							
ALT > 3 × ULN	0	0	0	0	0	0	0
Renal function							
Normal (eGFR ≥ 90 mL/min/1.73 m^2^)	38 (76.0)	32 (64.0)	30 (60.0)	37 (74.0)	30 (60.0)	34 (68.0)	30 (60.0)
Mild (eGFR 60–89 mL/min/1.73 m^2^)	10 (20.0)	14 (28.0)	16 (32.0)	9 (18.0)	14 (28.0)	11 (22.0)	15 (30.0)
Moderate (eGFR 30–59 mL/min/1.73 m^2^)	2 (4.0)	4 (8.0)	5 (10.0)	4 (8.0)	6 (12.0)	5 (10.0)	4 (8.0)
Severe (eGFR 15–29 mL/min/1.73 m^2^)	0	0	0	0	0	0	0
Kidney failure (eGFR < 15 mL/min/1.73 m^2^)	0	0	0	0	0	0	0
Microalbuminuria							
At baseline without ACE inhibitors and/or ARBs	2 (4.0)	2 (4.0)	0	1 (2.0)	1 (2.0)	2 (4.0)	4 (8.0)
At 2 years after without ACE inhibitors and/or ARBs	2 (4.0)	2 (4.0)	0	1 (2.0)	1 (2.0)	1 (2.0)	1 (2.0)
At baseline with ACE inhibitors and/or ARBs	8 (16.0)	17 (34.0)	11 (22.0)	15 (30.0)	13 (26.0)	12 (24.0)	17 (34.0)
At 2 years after with ACE inhibitors and/or ARBs	8 (16.0)	20 (40.0)	14 (28.0)	17 (34.0)	14 (28.0)	15 (30.0)	18 (36.0)
Macroalbuminuria	0	0	0	0	0	0	0
CK level							
>10 × ULN	0	0	0	0	0	0	0
